# A 43 Bp-Deletion in the *F3′H* Gene Reducing Anthocyanins Is Responsible for Keeping Buds Green at Low Temperatures in Broccoli

**DOI:** 10.3390/ijms241411391

**Published:** 2023-07-13

**Authors:** Huifang Yu, Jiansheng Wang, Yusen Shen, Xiaoguang Sheng, Ranjan Kumar Shaw, Ferdinando Branca, Honghui Gu

**Affiliations:** 1 Institute of Vegetable, Zhejiang Academy of Agricultural Sciences, Hangzhou 310021, China; 2 Department of Agriculture, Food and Environment, University of Catania, 95123 Catania, Italy

**Keywords:** BSA, mapping, low temperature, anthocyanins, F3′H

## Abstract

Most broccoli cultivars or accessions exhibit green buds under appropriate growth conditions, which turn purple at cold temperatures. However, certain cultivars consistently maintain green buds both during normal growth and at cold temperatures. In this study, we used BSA-seq (bulked segregation analysis-sequencing), along with fine mapping and transcriptome analysis to identify a candidate gene (flavonoid 3′-hydroxylase, *F3′H*) responsible for reducing anthocyanin accumulation in the mutant *GS* and HX-16 broccoli (*Brassica oleracea* L. var. *italica*), which could retain green buds even at low temperatures. A 43-bp deletion was detected in the coding sequence (CDS) of the *F3′H* gene in HX-16 and the mutant *GS*, which significantly decreased *F3′H* expression and the accumulation of cyanidin and delphinidin in the mutant *GS*. Furthermore, the expression of *F3′H* was upregulated at low temperatures in the wild line PS. Our results demonstrated the efficacy of utilizing the 43-bp InDel (Insertion–Deletion) in predicting whether buds in *B. oleracea* L. will turn purple or remain green at cold temperatures across forty-two germplasm materials. This study provides critical genetic and molecular insights for the molecular breeding of *B. oleracea* and sheds light on the molecular mechanisms underlying the effect of low temperatures on bud color in broccoli.

## 1. Introduction

Anthocyanins are flavonoids that exhibit a wide distribution in plants, endowing them with colors ranging among orange, red, purple, and blue [1,2,3]. Moreover, these compounds play a crucial role in plant defense against various abiotic and biotic stresses, such as cold, intense sunshine, and microbe infection [4]. Despite the identification of over 600 anthocyanins, they can be categorized into only six core structures: cyanidin, delphinidin, malvidin, pelargonidin, peonidin, and petunidin [5]. While anthocyanins are prevalent in plants, they are not indispensable. Their biosynthesis, which is part of the flavonoid biosynthesis pathway, has been extensively studied using relevant mutants, especially in the model plant *Arabidopsis thaliana* [3,6,7]. In *Arabidopsis*, many genes associated with anthocyanin biosynthesis were identified using mutant lines that exhibit a *transparent testa* (*tt*) phenotype [8].

The biosynthesis of anthocyanins starts with phenylalanine, which undergoes a series of enzymatic conversions, including phenylalanine ammonia lyase (PAL), cinnamate 4-hydroxylase (C4H), and 4-coumarate CoA ligase (4CL), ultimately leading to the formation of 4-coumaroyl CoA within the general phenylpropanoid pathway [6,9].

Subsequently, malonyl-CoA and 4-coumaroyl CoA are enzymatically catalyzed by a series of enzymes, including chalcone synthase (CHS), chalcone isomerase (CHI), and flavone 3-hydroxylase (F3H) to produce dihydroflavonols. These dihydroflavonols are further converted into anthocyanidins through a series of enzymatic reactions involving F3′H (flavonoid 3′-hydroxylase), DFR (dihydroflavonol 4-reductase), and ANS (anthocyanidin synthase)/LDOX (leucoanthocyanidin dioxygenase). Dihydroflavonols can also be oxidized to flavonols by FLS (flavonol synthase). Anthocyanidins are inherently unstable and are often stabilized through processes such as glycosylation, methylation, and/or acylation [6,9]. Additionally, the biosynthesis of anthocyanins is regulated by various factors, including transcription factors (R2R3MYB and bHLH), WD repeat proteins (WDR), epigenetic modification, and environmental factors such as light, temperature, sugar, hormones, fertilizer, drought, infection of pathogens, etc. [6,9,10,11,12,13,14]. Environmental factors play a critical role in regulating the biosynthesis of anthocyanin by modifying the expression of its structural or regulatory genes. The accumulation of anthocyanins in apple skin is induced by low temperatures and intense sunlight, which enhance the expression of *CHS*, *ANS*, and *UFGT* [15]. Conversely, light induces anthocyanin accumulation in apple fruit skin through MdSIZ1 modification of MdMYB1 [16,17]. In *Arabidopsis*, CBF1 (CRT/DRE-binding factor1, also known as DREB1B) regulates anthocyanin rhamnosyl transferases, UGT79B2 and UGT79B3, thereby increasing low-temperature tolerance via modulating anthocyanin accumulation [8]. The structural genes involved in anthocyanin biosynthesis, such as *CHS*, *F3′H*, *F3H*, and *UFGT*, as well as the transcript factor *MYB*, are also regulated by low temperatures [18,19]. However, the impact of low temperatures on anthocyanin biosynthesis varies among different species [20,21]. While low temperature induces the accumulation of anthocyanins in plants like *Arabidopsis* [8], apples [15,21], and kale [19], it exerts the opposite effect on anthocyanin accumulation in peaches and strawberries [20,22]. Anthocyanin decoration also plays a vital role in enhancing environmental stress tolerance [5].

Several cultivars or accessions of *Brassica* spp. exhibit purple leaves, bulbs, or curds under normal growth conditions [23,24]. In purple cauliflower, the activation of the *R2R3 MYB* transcription factor is triggered by a Harbinger DNA transposon insertion in its regulatory region, leading to the activation of anthocyanin structural genes, such as *F3′H*, *DFR*, and *ANS* and resulting in pigment accumulation [23]. Similarly, a 7600 bp CACTA-like transposon and 1-bp insertion in the *BoMYB2* promoter region have been identified in purple kohlrabi and purple cabbage, respectively [24]. While most broccoli cultivars or accessions do not exhibit a purple or red bud trait under optimal growth temperatures, they turn purple under abiotic stress environments, such as low temperatures and intense sunlight. In contrast, some broccoli plants maintain green buds even at cold temperatures. In this study, we investigated the genetic control of green/purple buds in broccoli (*Brassica oleracea* L. var. *italica*) under low temperature conditions. We constructed a segregating population consisting of 987 individuals, performed fine-mapping of the trait, and cloned the candidate gene. Additionally, we investigated another segregating population with the same trait, as well as other accessions in *Brassica*, using a marker based on the candidate gene. This study provides crucial information for the genetic breeding of *B. oleracea* and sheds light on the genetic mechanisms underlying the effect of low temperatures on bud color in broccoli.

## 2. Results

### 2.1. Anthocyanin Contents

**Figure 1:**
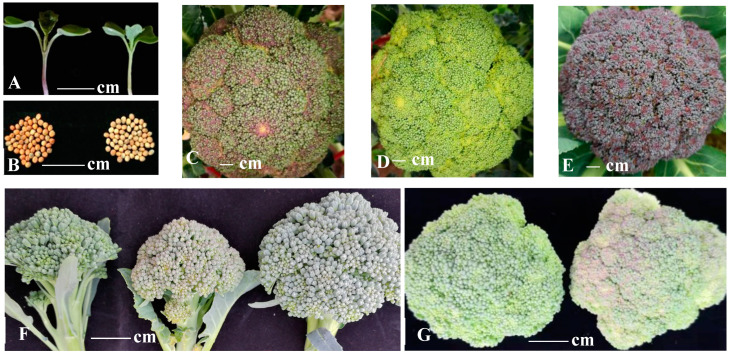
Phenotype of the seedlings, seeds, and bud heads in different broccoli materials. (**A**,**B**): seedlings and seeds of the wild line PS (left) and the mutant GS (right), respectively. (**C**–**E**): bud heads from the wild line PS, the mutant GS, purple broccoli (PB). (**F**): the paternal line HX-16, the maternal line HX-11, and F_1_ at low temperature, respectively. (**G**): the buds of representative individuals in the BC_2_F_2_ population.

The mutant GS and the wild DH line PS showed different colors in their seeds and seedling hypocotyls (Figure 1A,B), as well as different bud colors at low temperatures (Figure 1C,D). To assess the levels of anthocyanin and anthocyanidins in the buds of GS and PS during cold winter temperatures (between 0 and 5 °C), we conducted tests for total anthocyanin content and six types of anthocyanidins. In this test experiment, a purple broccoli accession (PB) was used as a reference material. As expected, PB displayed the highest total anthocyanin content among the three materials, while GS exhibited approximately 66% of the total anthocyanin content compared to PS (Table 1). The contents of cyanidin and delphinidin in GS were 45% and 26% lower than those in PS, respectively. The malvidin content was very low even in the purple broccoli and remained similar in PS and GS. Pelargonidin, peonidin, and petunidin were not detected in any of the broccoli samples (Table 1).

### 2.2. Mapping of the Green/Purple Bud Trait at Cold Temperatures in Broccoli

HX-11 and HX-16, whose buds turn purple and stay green at cold temperatures, respectively, were selected to construct a BC_2_F_2_ segregating population in order to map the trait (Figure 1F). Bulked segregant analysis (BSA) was employed to map the target region of interest associated with the trait. In the BC_2_F_2_ segregating population, which consisting of 987 individuals, 252 and 735 plants exhibited green and purple buds at low temperatures, respectively, conforming to a segregation ratio of 1:3. This result indicated that a single locus was responsible for controlling the trait.

**Figure 2:**
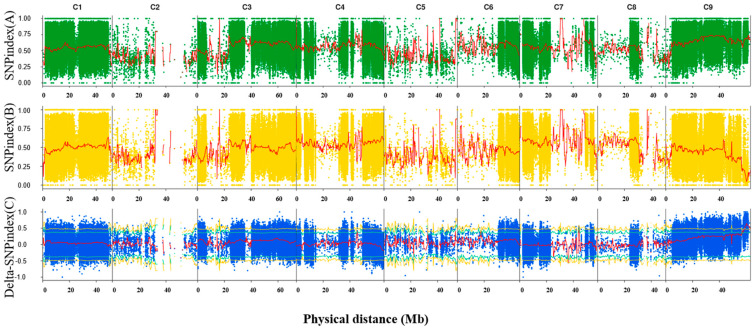
The result of BSA of broccoli buds color at low temperature. (**A**) Grahps of SNP index of the green bud pool, (**B**) graphs of the SNP index of the purple bud pool and (**C**) the ΔSNP index values used for the association analysis.

To identify the locus associated with green/purple buds at low temperatures, 30 individuals with green buds at cold temperatures were selected from the BC_2_F_2_ segregating population to create a green pool (G-pool), along with another 30 individuals with purple buds at low temperatures forming the purple pool (P-pool). These two pools were utilized to conduct BSA-seq. The genomic DNA was sequenced using the Illumina NovaSeq platform, yielding 63.86 Gb of clean data (Table S1). The obtained data exhibited high quality, with low N rates (0) and high scores of Q20 (96.66–98.13) and Q30 (91.06–94.51) (Table S1). Through filtering the raw data, high-quality data were generated, with over 99.34–99.59% of the data being successfully mapped to the reference genome, HDEM (http://www.genoscope.cns.fr/externe/plants/chromosomes.html (accessed on 1 March 2021)) (Table S2). The average sequencing depths of maternal, paternal, G-pool, and P-pool were 22.58, 22.87, 27.30, and 27.82, respectively, with mapping coverage ranging from 86.50–89.03% (Table S3). In the parental lines, 2,334,791 homozygous SNPs identical to the reference genome were called (ref), and 3,120,872 homozygous SNPs were different from the reference genome (alt) (Table S4). Additionally, 322,714 homozygous InDels identical to the reference genome and 615,872 homozygous InDels differing from the reference genome were called in the parental lines (Table S5). The SNP-index distribution in the genomes of the G-pool and P-pool was analyzed and is shown in Figure 2A,B. The subtraction of the two SNP indexes resulted in the Delta (Δ) SNP index (Figure 2C). The candidate region associated with the target trait was chosen based on the Δ SNP index value, and the peak area above the 99% confidence coefficient was identified. Consequently, the 4.6 Mb region between 58,700,000 and 63,300,000 bp on chromosome 9 was identified as the target region for the green/purple-bud trait at cold temperatures.

### 2.3. Fine Mapping

Thirty-one KASP primers were designed based on the SNP information within the target region spanning C9: 58,700,000–63,300,000 of the parent lines. Initially, the primers were screened using the parental lines and a random sample of 20 plants selected from the 987 BC_2_F_2_ individuals. As a result, 30 KASP markers were identified suitable for further testing on the remaining individuals in the segregating population. All 987 plants in the BC_2_F_2_ population were phenotyped and genotyped using four KASP primers located on either side of the target region to identify the recombinant individuals. Finally, 96 individuals with recombined chromosome segments within the target region were chosen for fine mapping. These recombined individuals were genotyped using the remaining 26 KASP markers. The phenotypic and genotypic (with 30 KASP markers) results of the recombined individuals are presented in Table S6. The genotypic data were arranged from top to bottom based on ascending order of the 30 KASP markers, which were lined from left to right by physical location in the reference genome as described in the materials and methods. These data strongly supported the mapping result, indicating a close association between the target region and bud color at low temperatures.

Furthermore, based on the genotypic and phenotypic data of the recombinants with chromosome segment exchange within the target region, the trait was fine-mapped to a region of approximately 271 Kb between bropK17 (C9: 59,974,705) and bropK19 (C9: 60,246,054) (Table S6). 

Within the range of C9: 59,974,705–60,246,054, an additional 6 KASP primers were developed based on the relative SNP information. Subsequently, 19 individual plants with recombined chromosome segments within this range were genotyped using the 6 KASP markers. The phenotyping and genotyping analysis of these 19 recombinants further narrowed down the target area to a 241Kb range between bropK41 (C9: 59,998,177) and bropK43 (C9: 60,239,356) (Table S7). Additionally, based on the InDel information within the region of C9: 59,998,177–60,239,356 in the parental lines, 6 more KASP primers were developed and used to genotype the 12 individuals with the recombined genome in this area (Table S8). Finally, the target gene was fine-mapped to a region of about 240 kb between bropK47 and bropK43, located between C9: 59,999,594 and 60,239,356 (Table S8). 

### 2.4. The Application of the KASP Markers

The fine-mapping results revealed a close association between the target gene and six KASP markers (bropsK49, bropsK18, bropsK51, bropsK52, bropsK42, and bropsK43) and any one of them could potentially be part of the target gene. To validate the accuracy of these six markers, 92 individuals from the F_2_ segregation population, derived from the mutant GS and the wild line PS, were screened. The genotyping results of five KASP markers (bropsK18, bropsK51, bropsK52, bropsK42, and bropsK43) were in agreement with the phenotypic data by approximately 99%, while the genotyping result of bropsK49 showed 100% accordance with the phenotypic data (Table S9). This demonstrates the effectiveness of the six KASP markers located within the region of C9: 59,999,594 to 60,239,356 in genotyping each plant in the new population (Table S9). Based on the genotypic and phenotypic data of the F_2_ individuals, the genomic location range associated with bud color at low temperatures was further narrowed down to 229.649 Kb, located between bropK47 and bropK18, i.e., C9: 59,999,594–60,229,243. It is likely that the target gene is located within the region of bropsK49.

Additionally, to evaluate the usability of the six KASP markers, 42 germplasm materials in *B. oleracea* L. were screened (Table 2). Among the 42 accessions, 12 lines keep green buds at cold temperatures, while 30 lines turn purple. Out of the six KASP markers, bropsK18 and bropsK49 displayed a co-segregation of over 95% between the genotyping and phenotyping data (Table S10). Among the 42 germplasm lines, 40 lines displayed consistent results for both bropsK18 and bropsK49, with the genotypic data in agreement with the phenotyping data. However, two lines (1947 and K2145), whose buds stay green at low temperatures, lacked a 43-bp deletion in bropsK49 and exhibited inconsistent results between genotyping and phenotyping. Specifically, out of the 12 lines with green buds at low temperature, 10 lines contained the 43-deletion in *F3′H*, while the remaining two lines did not contain the 43-bp deletion in *F3′H* (Table S10).

### 2.5. Expression Profile of Anthocyanin-Related Genes in the Mutant and the Wild Line

**Figure 3:**
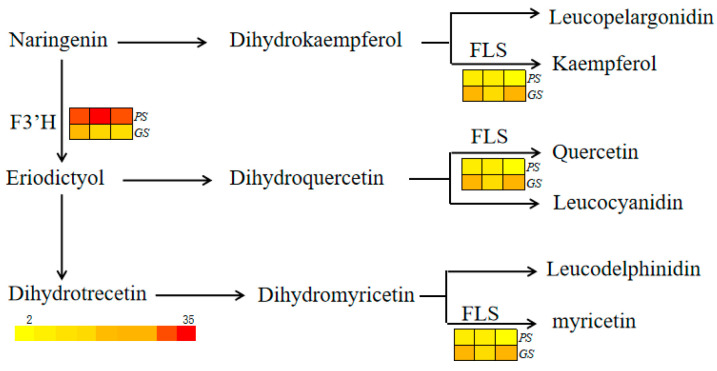
The genes involved in the anthocyanin biosynthesis pathway were differentially expressed at low temperatures in the wild line PS and the mutant line GS. The color gradient from red to yellow shows the relative expression levels of each gene, with red representing high expression and yellow representing low expression.

A total of 1729 DEGs (differentially expressed genes) were identified between the green buds of GS and the purple buds of PS. Among the 53 genes within the 229.6 Kb range on chromosome C9 (between 59,999,594 and 60,229,243 bp) in the broccoli reference genome (Table S11), only one gene, *BolC9t59639H* (*F3′H*), showed differential expression between the mutant GS and the wild line PS. The expression level of *BolC9t59639H* (* F3′H*) in the buds of the mutant GS was one-third of that in the buds of the wild line PS at low temperatures. Furthermore, the KEGG database analysis revealed 43 genes related to anthocyanin biosynthesis and the regulation pathway that were expressed in the buds of broccoli PS and GS under low temperature conditions. In addition to *BolC9t59639H* (*F3′H*), the expression level of *BolC3t18539H* (*FLS*) differed in both buds, with approximately 1.5 times higher expression in the buds of the mutant GS compared to the wild line (Figure 3). According to KEGG Pathway enrichment analysis, BolC9t59639H (F3′H, EC: 1.14.14.82) is involved in the biosynthesis of cyanidins and delphinidins, while BolC3t18539H (FLS, EC: 1.14.20.6) plays a role in flavone and flavonol biosynthesis (https://www.kegg.jp/pathway/map=map00941&keyword=flavone (accessed on 8 June 2023)).

### 2.6. Validation of Anthocyanin-Related Genes by qRT-PCR

**Figure 4:**
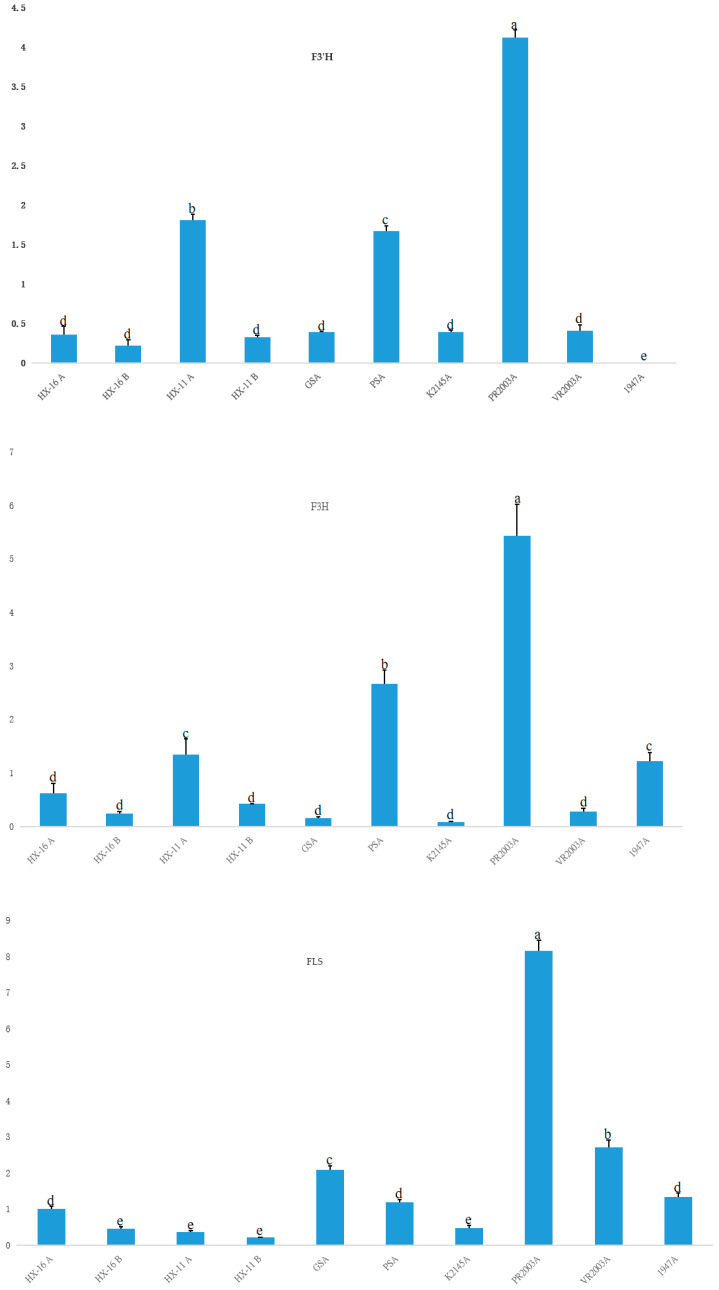
The expression levels of F3′H, F3H, and FLS in different materials by qRT-PCR. A and B after the material codes are for after and before experiencing cold temperatures, respectively. Different letters on the top of columns represent variance analysis (*p* ≤ 0.05). Error bars are for standard deviation.

The expression levels of *F3′H*, *F3H,* and *FLS* were validated using qRT-PCR with buds from HX-16 and HX-11 before and after exposure to cold temperatures, as well as buds from GS, PS, K2145, PR2003, VR2003, and 1947 at cold temperatures (Figure 4). Cold temperature was found to increase the expression of *F3′H*, *F3H*, and *FLS* in the buds of HX-16 and HX-11. Among the eight accessions, PR2003, a purple cauliflower line, exhibited the highest expression levels of these three genes in its buds.

The expression of *F3′H* in HX-16, GS, K2145, and 1947, which retained green buds at cold temperatures, was lower compared to HX-11, PS, PR2003, and VR2003, all of which displayed purple buds at low temperatures. Notably, 1947, which exhibited green buds at low temperatures and lacked the 43-bp deletion, showed the lowest expression of *F3′H*. On the other hand, *F3H* and *FLS* showed higher expression levels in 1947.

### 2.7. cDNA and Predicted Amino Acid Sequences of the F3′H Gene in HX-11 and HX-16

**Figure 5:**
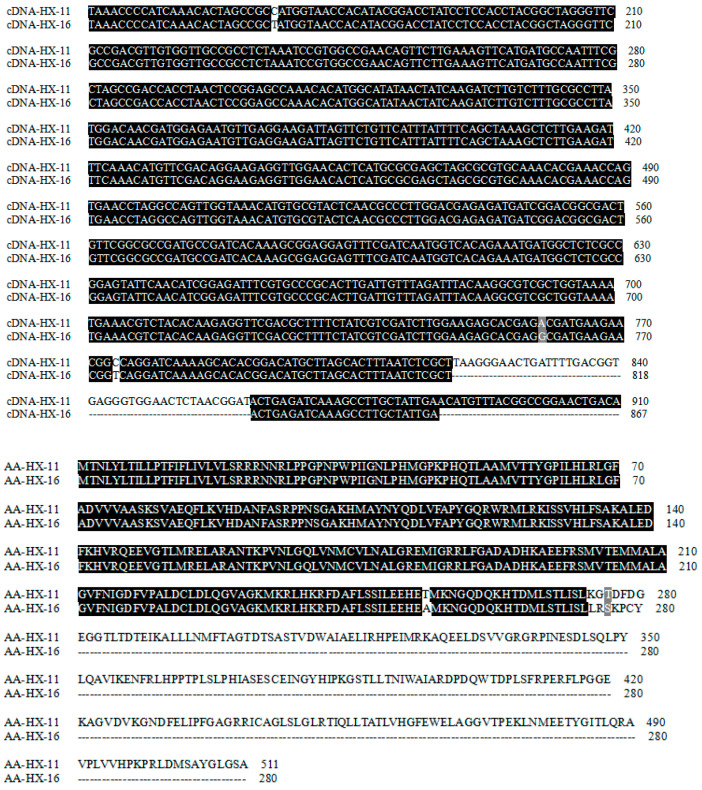
The alignment of partial cDNA and predicted amino sequences of the *F3′H* gene in HX-11 and HX-16, respectively. In HX-16, a 43-bp deletion was identified in the *F3′H* cDNA sequence and this deletion results in an early appearance of the stop codon (TGA). The black highlights are for the same amino acid sequence, and the grey for the similar amino acids.

The complete cDNA sequences of *F3′H* in HX-11 and HX-16 were determined to be 1536 bp and 843 bp in length, respectively (Table S12). A comparison between HX-11 and HX-16 revealed three single nucleotide polymorphisms (SNPs) and a 43-bp insertion–deletion (InDel) variation, as depicted in Figure 5. The predicted amino acid sequences of *F3′H* in HX-11 and HX-16 consisted of 512 aa and 281 aa, respectively. The predicted amino acid sequence of *F3′H* in HX-16 is shorter than that of HX-11 due to a 43-bp deletion, which resulted in an early appearance of the stop codon (TGA) in HX-16.

## 3. Discussion

Anthocyanins are characterized by six core structures: cyanidin, delphinidin, malvidin, pelargonidin, peonidin, and petunidin [22]. The genus- and species-specificity of anthocyanins is a result of the substrate specificity of key enzymes in the biosynthetic pathway, which compete among themselves on branch nodes [22]. In this study, we detected only cyanidin, delphinidin, and malvidin in three broccoli accessions, with no presence of pelargonidin, peonidin, or petunidin. Cyanidin was found to be the predominant anthocyanin in purple broccoli, exhibiting similar levels to delphinidin in PS, and had less content than delphinidin in GS. These results were consistent with a previous study [25] and demonstrated the species-specific characteristics of anthocyanins in broccoli. The vivid purple color of buds in purple broccoli appears to be attributable to the accumulation of cyanidins, with low temperatures primarily promoting their accumulation in PS. Conversely, GS displayed the most significant decrease in cyanidin content compared to PS. In *Arabidopsis*, mutants lacking specific steps in anthocyanin biosynthesis have been identified, and the corresponding mutants exhibit transparent testa due to the lack of particular steps in the anthocyanin biosynthesis pathway [8]. For instance, the *Arabidopsis tt7* mutant lacks a flavonoid 3′-hydroxylase and does not accumulate anthocyanins. However, transgenic *Arabidopsis tt7* seedlings expressing apple *MdF3′H* regain red color pigmentation in seed coats and accumulate both pelargonidin and cyanidin under nitrogen-deficient conditions [26]. This suggests that MdF3′H affects the accumulation of pelargonidin and cyanidin in *Arabidopsis*. According to the anthocyanin biosynthetic pathway, *F3′H* encodes flavonoid 3′-hydroylase, which catalyzes the conversion of kaempferol into quercetin and dihydrokaempferol into dihydroquercetin and is the key gene for cyanidin and delphinidin biosynthesis [26,27]. Therefore, the green-bud trait observed at low temperatures in GS and HX-16 could be due to the 43-bp deletion in the *F3′H* CDS, leading to decreased accumulation of cyanidins and delphinidins.

The genotyping results of the BC_2_F_2_ segregating population, comprising 987 individuals, using the marker bropsK18, were consistent with the phenotyping data. However, when genotyping the F_2_ segregating population derived from PS and GS with the same marker, the results were not consistent with the phenotyping data. On the other hand, the phenotyping results were completely consistent with the genotyping results of marker bropsK49, which was designed based on the DNA sequences of the BolC9t59639H (*F3′H*) gene. This gene contained a 43-bp InDel in the second exon within the target region between HX-11 and HX-16. These findings suggest that the presence of the 43-bp deletion in *BolC9t59639H* (*F3′H*) is responsible for the green-bud trait observed at low temperatures in HX-16 and the mutant GS.

In both the mutant GS and wild line PS, only two genes related to anthocyanin biosynthesis showed differential expression in the buds. Specifically, *F3′H* was expressed at lower levels, while *FLS* was expressed at higher levels in the mutant. Although *F3′H* and *FLS* are involved in the biosynthesis pathway of anthocyanin, flavone, and flavonol, respectively, they share certain substrates. For instance, dihydroflavonol, a substrate for *F3′H*, can also be used by FLS [28,29]. FLS catalyzes dihydroflavonol to flavonol, while *F3′H* hydroxylates the 3′-position of the B-ring in flavanone, flavonol, and dihydroflavonol [29,30]. Hence, if the expression of *F3′H* decreases, the competitive relationship for the same substrate might cause an increase in the expression of *FLS*. In the mutant, the expression profiles of *F3′H* and *FLS* are likely a result of this competitive relationship for the same substrate.

The accumulation of anthocyanins is affected by ambient temperature in plants, and usually, low temperatures stimulate the expression of the genes involved in the anthocyanin biosynthesis pathway, including *PAL*, *C4H*, *4CL*, *CHS*, *CHI*, *F3H*, *F3′H*, *DFR*, *ANS,* and *UGT75C1*, as well as regulating factors like *MYB* [31,32]. However, some unpublished data in broccoli showed a reduction in *ANS* expression at low temperatures, which seems contradictory to pigment accumulation since *ANS* is a crucial enzyme that catalyzes the production of anthocyanin monomers from leucoanthocyanidins [28,33]. Additionally, *ANS* not only synthesizes anthocyanins, but also possesses the same activity as *FLS* [34,35,36]. Moreover, in *Arabidopsis*, *AtFLS1* and/or *AtANS* have partial activity of the *F3H* enzyme [37], and *FLS* can partially complement *ANS* in Arabidopsis *tt6* mutants [36]. *FLS* and *ANS* share highly similar polypeptides and both can interact with leucoanthocyanidins [38].

Low temperature primarily induces anthocyanin accumulation in most plants [8,15,18,20]. However, due to the species-specificity and complexity of anthocyanins, in certain plants, low temperatures can reduce their accumulation [20,22]. Therefore, although the biosynthesis pathway of anthocyanins in *Arabidopsis* is well understood, the specificity of anthocyanin biosynthesis and regulation in broccoli remain unknown. In this investigation, the 43-bp deletion in *F3′H* CDS was found to decrease gene expression and ultimately reduce the accumulation of cyanidin and delphinidin in certain broccoli cultivars and accessions, which keep green buds at cold temperatures. However, the accuracy of the bropsK49 marker in *Brassica* germplasm accessions was 96 percent, indicating that apart from the 43-bp deletion in *F3′H*, there might be different mutations occurring in *F3′H* or other relative factors influencing anthocyanin accumulation in *Brassica* at low temperatures. Other studies have demonstrated the involvement of a 68-bp deletion in the DNA sequences of *F3′H* and a 1-bp insert in the exon of *DFR*, leading to green buds and green leaves in broccoli and kale, respectively [39,40].

Typically, an InDel mutation in a gene’s CDS may not change the expression level of the gene, but instead affect its function. In the case of *F3′H* in GS and HX-16, the downregulation of its expression levels may be attributed to the alteration of the protein’s amino acid sequence and structure caused by the 43-bp deletion. This alteration could prevent the binding of proteins that interact with *F3′H* and activate its function. Alternatively, the 43-bp deletion may not be the sole cause of this effect since lines 1947 and K2145, which lack the deletion, exhibit minimal or lower expression of *F3′H*. It is also possible that the promoters of *F3′H* are responsible for this effect.

## 4. Materials and Methods

### 4.1. Construction of F_2_ and Backcross Populations

The broccoli DH lines HX-16 and HX-11, which have green and purple buds at low temperatures, respectively, were selected as paternal and maternal lines, respectively, and were crossed to develop the F_1_ hybrid. The BC_1_ population was obtained by pollinating the F_1_ hybrid with HX-16. In the BC_1_ population, individuals with purple buds at low temperatures were selected to cross with HX-16 again, producing the BC_2_ population. In the BC_2_ population, individuals with purple buds at low temperatures were selected and selfed to construct the BC_2_F_2_ segregation population. An F_2_ segregation population was obtained by selfing the hybrid F_1_ derived from the mutant GS with green buds and the wild line PS with purple buds at low temperatures.

Plug-seedlings were obtained and moved to a tunnel greenhouse at Yangdu Experimental Farm of the Zhejiang Academy of Agricultural Sciences in the autumn of 2017 (HX-11, HX-16, F_1_, the mutant GS, and wild DH line PS), 2018 (HX-11, HX-16, F_1_, BC_1_, the mutant GS, and the wild DH line PS), 2019 (HX-11, HX-16, F_1_, BC_1_ and BC_2_, the hybrid F_1_ of the mutant GS and the wild lines PS, and germplasm materials in *Brassica oleracea* (Table 2)), 2020 (HX-11, HX-16, F_1_, BC_2_F_2_, the mutant GS, the wild line PS, and the F_2_ segregation population,) and 2021 (HX-11, HX-16, F_1_, the mutant GS, the wild lines PS, and purple broccoli (PB)). Phenotyping of the buds in the segregation populations, respective parents, and the germplasm materials was conducted after exposure to cold air (0–5 °C) for 10 days, as the temperature of this range could make the buds of ordinary broccoli purple.

### 4.2. Anthocyanin Extraction and Measurement

The experiment utilized buds from three different broccoli lines: the mutant GS, the wild line PS, and the purple broccoli PB, which were grown at low temperature, as shown in Figure 1C–E. Total anthocyanins were extracted from the buds using a 0.1 mol/L ethanol hydrochloride solution and then detected using a UV/VIS spectrophotometer. After hydrolyzing the anthocyanins with 37% HCl and boiling, the anthocyanidins were obtained. The content and constituents of the anthocyanidins were determined using HPLC-MS, following the method described by Zhang et al. in 2004 [41]. SPSS 21 was used to conduct one-way ANOVA analysis with LSD(L), Tukey s-b(K) and Waller–Duncan comparisons for the data.

### 4.3. Genomic DNA Extraction and Library Construction

Whole genomic DNA was extracted from frozen young leaves using the CTAB method [42] (Yu et al., 2019). Two pools were constructed, the P pool and G pool, representing the purple bud and green bud samples, respectively, with each pool containing DNA from 30 BC_2_F_2_ individuals. The quality of the DNA was assessed and the concentration was determined using electrophoresis on 0.8% agarose gel and a spectrophotometer (NanoDrop, Waltham, US, respectively. Sequencing libraries were prepared following the TruSeq DNA PCR-free prep kit instructions. The quality of libraries was tested using an Agilent High Sensitive DNA kit by Agilent Bioanalyzer, and the qualified libraries were sequenced using the Illumina NovaSeq platform. The raw sequencing data were filtered using fastp (v0.20.0) to discard those reads with low quality.

### 4.4. QTL Mapping

The high-quality reads were aligned to the reference genomic sequences (HDEM (http://www.genoscope.cns.fr/externe/plants/chromosomes.html, accessed on 1 March 2021) using BWA (0.7.12-r1039) [43]. Reads near InDel variant sites were realigned using the Indel Realigner of GATK to improve the precision of SNP acquisition. Precise and reliable SNPs were obtained by Unified Genotyper, and atypical SNPs were filtered out. The SNP-index was then calculated and mapped after SNP calling. The SNP-index in the p-pool and g-pool, as well as their subtraction, was mapped to show their distribution on the chromosome. The candidate region for the target trait was selected based on the subtraction value of the p-pool and g-pool. Accurate phenotyping is essential for precise QTL mapping, so the bud color trait was evaluated after exposure to cold for ten days.

### 4.5. KASP Primer Designing and Genotyping

KASP primers were designed based on the flanking sequences of the SNPs/InDels (Table S13). Genotyping of the BC_2_F_2_ and other populations was performed using the KASP platform (IntelliQube, LGC, Biosearch Technologies, Hoddesdon, UK) and followed the KASP genotyping protocol. The KASP reaction mixture, totaling 1.6 μL, included 5–10 ng of DNA sample, 1.74 μM of a common reverse primer, 0.69 μM of each Fam and Hex labeled forward primer, and 1x KASP Master Mix. The KASP protocol utilized a touchdown PCR method: step 1, 94 °C for 15 min; step 2 (touchdown), 94 °C for 20 s, 61 °C for 60 s (decreasing 0.6 °C per cycle) for 10 cycles; step 3, 94 °C for 20 s, 55 °C for 60 s for 26 cycles. The amplification products’ fluorescence signal was detected and classified.

### 4.6. Transcriptome Analysis

After exposure to cold air (0–5 °C), the buds of the mutant GS and the wild line PS were collected, frozen in liquid nitrogen, and then preserved in a −80 °C refrigerator. RNA was extracted from the samples using the Trizol Reagent (Invitrogen Life Technologies, Carlsbad, CA, USA). The quality of extracted RNA was tested by a NanoDrop spectrophotometer (Thermo Scientific, Waltham, MA, USA). Sequencing libraries were constructed using the TruSeq mRNA Sample Prep Kit (Illumina, San Diego, CA, USA). The mRNA was isolated from total RNA and fragmented to approximately 200–300 bp. cDNA was synthesized from the mRNA, and fragments of 300–400 bp were selected to construct the libraries. cDNA quality was evaluated on a Bioanalyzer 2100 system (Agilent, Santa Clara, CA, USA). The sequencing library was then sequenced on a Hiseq platform (Illumina, San Diego, CA, USA). Clean and high-quality reads were aligned to the reference genome (HDEM genome sequence) by HISAT2 2.0.5 with default parameters, and the expression level of each gene was calculated based on the alignment result. The aligned reads were assembled into transcript sequences. The read count value of each gene, representing the original gene expression, was calculated using HTSeq. The read counts were found to be positively correlated with the true gene expression level, sequence length, and sequencing depth. To ensure a meaningful comparison of gene expression levels across different genes and samples, we employed FPKM (fragments per kilo bases per million fragments) normalization. For paired-end sequencing, where two reads exist per fragment, FPKM counts only for the number of fragments from the two reads that can be compared to the same transcript. In reference transcripts, genes with FPKM > 1 are generally regarded as expressed. To determine differentially expressed genes (DEGs), we used DESeq and considered |log2FoldChange| > 1 and a significance *p*-value < 0.05 as the screening criteria for DEGs.

### 4.7. The CDS and Predicted Amino Acid Sequences of F3′H in HX-11 and HX-16

After exposure to cold air (0–5 °C), buds from HX-11 and HX-16 were collected, frozen in liquid nitrogen, and preserved in a −80 °C refrigerator. RNA was extracted from the samples using the Trizol Reagent (Invitrogen Life Technologies, Carlsbad, CA, USA). The cDNA was synthesized from mRNA using a TIANScript Kit (KR104) (Tiangen Biotech Co., Ltd., Beijing, China) following the instruction. The primers were designed based on the sequence of *F3′H* CDS in the HDEM reference genome to amplify the CDS sequences of HX-11 and HX-16 (Table S14). According to the complete CDS sequences in HX-11 and HX-16, their amino acid sequences of *F3′H* were predicted and aligned by BioXM 2.7.1.

### 4.8. qRT-PCR Analysis

The primers were designed using Primer-Blast on NCBI (Table S15). Buds were collected to extract total RNA using a polysaccharide and polyphenol total RNA isolation kit (RNAprep Pure Plant Kit; Tiangen, China). First-strand cDNA was synthesized using the PrimeScript RT reagent Kit. qRT-PCR was performed using the ABI StepOne Plus machine with SYBRVR Premix Ex TaqTM (TaKaRa, Shiga, Japan). The 25 µL reaction mixture contained 20–50 ng of first-strand cDNA products, 12.5 µL of 2 × SYBR Green PCR Master Mix (Applied Biosystems, Waltham, MA, USA), and 500 nmol of each primer. The PCR program included an initial step at 95 °C for 10 min followed by 40 cycles of 10 s at 95 °C, 60 °C for 15 s, and 72 °C for 30 s. *Boactin* was selected as the reference gene. Three biological replicates were performed for each sample. The relative expression of genes was determined using the 2^−ΔΔCt^ method, utilizing the Ct (threshold cycle) values of a gene and *Boactin* in a sample. Subsequently, the variance analysis was performed at a level of α = 0.05 (*p* ≤ 0.05) in Excel.

## Figures and Tables

**Table 1:** Total anthocyanins and six types of anthocyanidin contents.

	Total Anthocyanins C µg/g	CC µg/g	DC µg/g	MvC µg/g	PelC µg/g	PeoC µg/g	PtC µg/g
GS	12.123 ± 0.770 c	3.830 ± 0.420 b	4.190 ± 0.210 b	0.002 ± 0.011 b	0	0	0
PS	20.712 ± 0.378 b	6.980 ± 0.280 b	5.690 ± 0.090 b	0.002 ± 0.012 b	0	0	0
PB	103.138 ± 4.399 a	87.890 ± 4.480 a	9.110 ± 1.360 a	0.881 ± 0.323 a	0	0	0

Note: GS, PS, and PB are for the mutant GS, the wild line PS, and purple broccoli (PB), respectively. CC, DC, MvC, PelC, PeoC, and PtC stand for the content of cyanidin, delphinidin, malvidin, pelargonidin, peonidin, and petunidin, respectively. a, b, and c are analysis of variance (*p* < 0.05). The data were analyzed with a one-way ANOVA, followed by LSD(L), Tukey s-b(K), and Waller–Duncan analyses with SPSS 21 software.

**Table 2:** Germplasm materials in *Brassica oleracea*.

Material Codes	Species	Material Codes	Species	Material Codes	Species
1015	*B. oleracea* L. var. *italica*	SG1921	*B. oleracea* L. var. *italica*	SG1911	*B. oleracea* L. var. *italica*
1403	*B. oleracea* L. var. *italica*	SG1614	*B. oleracea* L. var. *botrytis*	SG1912-1	*B. oleracea* L. var. *italica*
1405-1	*B. oleracea* L. var. *italica*	51932-2	*B. oleracea* L. var. *italica*	SG1920	*B. oleracea* L. var. *italica*
1409	*B. oleracea* L. var. *italica*	51936-1	*B. oleracea* L. var. *italica*	SG2009-2	*Brassica oleracea* L. var. *kohlrabi*
1502-1	*B. oleracea* L. var. *italica*	51938-13	*B. oleracea* L. var. *italica*	SG2021	*B. oleracea* L. var. *acephala*
1610-4B	*B. oleracea* L. var. *italica*	PR2003	*B. oleracea* L. var. *botrytis*	UC001	*B. oleracea* L. var. *italica*
1910-1	*B. oleracea* L. var. *italica*	2035	*B. oleracea* L. var. *italica*	UC002	*B. oleracea* L. var. *botrytis*
2035	*B. oleracea* L. var. *italica*	1947	*B. oleracea* L. var. *italica*	UC003	*B. oleracea* L. var. *capitata*
2057-11	*B. oleracea* L. var. *italica*	VR2003	*B. oleracea* L. var. *botrytis*	UC012	*B. oleracea* L. var. *gongylodes*
5815-6A	*B. oleracea* L. var. *italica*	SG1401-1	*B. oleracea* L. var. *italica*	UC018	*B. oleracea* L. var. *gemmifera*
K2145	*B. oleracea* L. var. *acephala*	SG1913	*B. oleracea* L. var. *italica*	UC044	*B. oleracea* L. var. *italica*
K2150	*B. oleracea* L. var. *caulorapa*	SG1914-1	*B. oleracea* L. var. *italica*	UC019	*B. oleracea* L. var. *acephala*
2018K1M3	*B. oleracea* L. var. *acephala*	SG1917-2	*B. oleracea* L. var. *italica*	UC020	*B. oleracea* L. var. *gongylodes*
2018K1M5	*B. oleracea* L. var. *acephala*	SG1908	*B. oleracea* L. var. *italica*	UC030	*B. oleracea* L. var. *botrytis*

## Data Availability

All the raw sequencing reads in the manuscript have been deposited under NCBI BioProject accession numbers PRJNA980966 and PRJNA872779.

## Supplementary Materials

The supporting information can be downloaded at: https://www.mdpi.com/article/10.3390/ijms241411391/s1.

## References

[1] Springob K., Nakajima J., Yamazaki M., Saito K. (2003). Recent advances in the biosynthesis and accumulation of anthocyanins. Nat. Prod. Rep..

[2] Lepiniec L., Debeaujon I., Routaboul J.M., Baudry A., Pourcel L., Nesi N., Caboche M. (2006). Genetics and biochemistry of seed flavonoids. Annu. Rev. Plant Biol..

[3] Saito K., Yonekura-Sakakibara K., Nakabayashi R., Higashi Y., Yamazaki M., Tohge T., Fernie A.R. (2013). The flavonoid biosynthetic pathway in *Arabidopsis*: Structural and genetic diversity. Plant Physiol. Biochem..

[4] Tohge T., Fernie A.R. (2017). Leveraging natural variance towards enhanced understanding of phytochemical sunscreens. Trends Plant Sci..

[5] Saigo T., Wang T., Watanabe M., Tohge T. (2020). Diversity of anthocyanin and proanthocyanin biosynthesis in land plants. Curr. Opin. Plant Biol..

[6] Winkel-Shirley B. (2001). Flavonoid biosynthesis. A colorful model for genetics biochemistry, cell biology, and biotechnology. Plant Physiol..

[7] Grotewold E. (2006). The genetics and biochemistry of floral pigments. Annu. Rev. Plant Biol..

[8] Li P., Li Y., Zhang F., Zhang G., Jiang X., Yu H., Hou B. (2017). The Arabidopsis UDP-glycosyltransferases UGT79B2 and UGT79B3, contribute to cold, salt and drought stress tolerance via modulating anthocyanin accumulation. Plant J..

[9] Zhang Y., Butelli E., Martin C. (2014). Engineering anthocyanin biosynthesis in plants. Curr. Opin. Plant Biol..

[10] Espley R.V., Hellens R.P., Putterill J., Stevenson D.E., Kutty-Amma S., Allan A.C. (2007). Red colouration in apple fruit is due to the activity of the MYB transcription factor, MdMYB10. Plant J..

[11] Spelt C., Quattrocchio F., Mol J.N.M., Koes R. (2000). Anthocyanin1 of petunia encodes a basic helix–loop–helix protein that directly activates transcription of structural anthocyanin genes. Plant Cell.

[12] Lorenc-Kukula K., Jafra S., Oszmianski J., Szopa J. (2005). Ectopic expression of anthocyanin 5-o-glucosyltransferase in potato tuber causes increased resistance to bacteria. J. Agric. Food Chem..

[13] Wang Q., Wang Y., Sun H., Sun L., Zhang L. (2020). Transposon-induced methylation of the RsMYB1 promoter disturbs anthocyanin accumulation in red-fleshed radish. J. Exp. Bot..

[14] Yu L., Sun Y., Zhang X., Chen M., Wu T., Zhang J., Xing Y., Tian J., Yao Y. (2022). ROS1 promotes low temperature-induced anthocyanin accumulation in apple by demethylating the promoter of anthocyanin-associated genes. Hortic. Res..

[15] Ubi B.E., Honda C., Bessho H., Kondo S., Wada M., Kobayashi S., Moriguchi T. (2006). Expression analysis of anthocyanin biosynthetic genes in apple skin: Effect of UV-B and temperature. Plant Sci..

[16] Takos A.M., Jaffé F.W., Jacob S.R., Bogs J., Robinson S.P., Walker A.R. (2006). Light-induced expression of a *MYB* gene regulates anthocyanin biosynthesis in red apples. Plant Physiol..

[17] Zhou L., Li Y., Zhang R., Zhang C., Xie X., Zhao C., Hao Y. (2017). The small ubiquitin-like modifier E3 ligase MdSIZ1 promotes anthocyanin accumulation by sumoylating MdMYB1 under low-temperature conditions in apple. Plant Cell Environ..

[18] Zhang Y., Zheng S., Liu Z., Wang L., Bi Y. (2010). Both HY5 and HYH are necessary regulators for low temperature-induced anthocyanin accumulation in *Arabidopsis* seedlings. J. Plant Physiol..

[19] Zhang B., Hu Z., Zhang Y., Li Y., Zhou S., Chen G. (2012). A putative functional MYB transcription factor induced by low temperature regulates anthocyanin biosynthesis in purple kale (*Brassica Oleracea* var. *acephala* f. tricolor). Plant Cell Rep..

[20] Mao W., Han Y., Chen Y., Sun M., Feng Q., Li L., Liu L., Zhang K., Wei L., Han Z. (2022). Low temperature inhibits anthocyanin accumulation in strawberry fruit by activating FvMAPK3-induced phosphorylation of FvMYB10 and degradation of Chalcone Synthase. Plant Cell.

[21] Jiang H., Zhou L., Gao H., Wang X., Li Z., Li Y. (2022). The transcription factor MdMYB2 influences cold tolerance and anthocyanin accumulation by activating SUMO E3 ligase MdSIZ1 in apple. Plant Physiol..

[22] Zhu Y., Zhang B., Allan A.C., Lin-Wang K., Zhao Y., Wang K., Chen K., Xu C. (2020). DNA demethylation is involved in the regulation of temperature-dependent anthocyanin accumulation in peach. Plant J..

[23] Chiu L., Zhou X., Burke S., Wu X., Prior R.L., Li L. (2010). The Purple Cauliflower Arises from Activation of a MYB Transcription Factor. Plant Physiol..

[24] Yan C., An G., Zhu T., Zhang W., Zhang L., Peng L., Chen J., Kuang H. (2019). Independent activation of the BoMYB2 gene leading to purple traits in *Brassica oleracea*. Theor. Appl. Genet..

[25] Moreno D.A., Perez-Balibrea S., Ferreres F., Gil-Izquierdo A., Garcia-Viguera C. (2010). Acylated anthocyanins in Broccoli sprouts. Food Chem..

[26] Han Y., Vimolmangkang S., Soria-Guerra R.E., Rosales-Mendoza S., Zheng D., Lygin A.V., Korban S.S. (2010). Ectopic Expression of Apple *F3′H* Genes Contributes to Anthocyanin Accumulation in the Arabidopsis *tt7* Mutant Grown Under Nitrogen Stress. Plant Physiol..

[27] Seitz C., Ameres S., Forkmann G. (2007). Identification of the molecular basis for the functional difference between flavonoid 3′-hydroxylase and flavonoid 3′,5′-hydroxylase. FEBS Lett..

[28] Wang Y., Shi Y., Li K., Yang D., Liu N., Zhang L., Zhao L., Zhang X., Liu Y., Gao L. (2021). Roles of the 2-Oxoglutarate-Dependent Dioxygenase Superfamily in the Flavonoid Pathway: A Review of the Functional Diversity of F3H, FNS I, FLS, and LDOX/ANS. Molecules.

[29] Guo L., Gao L., Ma X., Guo F., Ruan H., Bao Y., Xia T., Wang Y. (2019). Functional analysis of flavonoid 3′-hydroxylase and flavonoid 3′,5′-hydroxylases from tea plant (*Camellia sinensis*), involved in the B-ring hydroxylation of flavonoids. Gene.

[30] Jia Y., Li B., Zhang Y., Zhang X., Xu Y., Li C. (2019). Evolutionary dynamic analyses on monocot flavonoid 3′-hydroxylase gene family reveal evidence of plant-environment interaction. BMC Plant Biol..

[31] Dai Y., Zhang L., Sun X., Li F., Zhang S., Zhang H., Li G., Fang Z., Sun R., Hou X. (2022). Transcriptome analysis reveals anthocyanin regulation in Chinese cabbage (*Brassica rapa* L.) at low temperatures. Sci. Rep..

[32] Li C., Yu W., Xu J., Lu X., Liu Y. (2022). Anthocyanin Biosynthesis Induced by MYB Transcription Factors in Plants. Int. J. Mol. Sci..

[33] Saito K., Kobayashi M., Gong Z., Tanaka Y., Yamazaki M. (1999). Direct evidence for anthocyanidin synthase as a 2-oxoglutaratedependent oxygenase: Molecular cloning and functional expression of cDNA from a red forma of *Perilla frutescens*. Plant J..

[34] Turnbull J.J., Sobey W.J., Aplin R.T., Hassan A., Firmin J.L., Schofield C.J., Prescott A.G. (2000). Are anthocyanidins the immediate products of anthocyanidin synthase?. Chem. Commun..

[35] Wellmann F., Griesser M., Schwab W., Martens S., Eisenreich W., Matern U., Lukacin R. (2006). Anthocyanidin synthase from Gerbera hybrida catalyzes the conversion of (+)-catechin to cyanidin and a novel procyanidin. FEBS Lett..

[36] Wilmouth R.C., Turnbull J.J., Welford R.W., Clifton I.J., Prescott A.G., Schofield C.J. (2002). Structure and mechanism of anthocyanidin synthase from *Arabidopsis thaliana*. Structure.

[37] Owens D.K., Crosby K.C., Runac J., Howard B.A., Winkel B.S. (2008). Biochemical and genetic characterization of Arabidopsis flavanone 3β-hydroxylase. Plant Physiol. Biochem..

[38] Turnbull J.J., Nakajima J.I., Welford R.W., Yamazaki M., Saito K., Schofifield C.J. (2004). Mechanistic studies on three 2-oxoglutaratedependent oxygenases of flavonoid biosynthesis: Anthocyanidin synthase, flavonol synthase, and flavanone 3 beta-hydroxylase. J. Biol. Chem..

[39] Liu C., Yao X., Li G., Huang L., Wu X., Xie Z. (2021). Identifification of Major Loci and Candidate Genes for Anthocyanin Biosynthesis in Broccoli Using QTL-Seq. Horticulturae.

[40] Tang Q., Tian M., An G., Zhang W., Chen J., Yan C. (2017). Rapid identification of the purple stem (Ps) gene of Chinese kale (*Brassica oleracea* var. *alboglabra*) in a segregation distortion population by bulked segregant analysis and RNA sequencing. Mol. Breed..

[41] Zhang Z., Kou X., Fugal K., Mclaughlin J. (2004). Comparison of HPLC Methods for Determination of Anthocyanins and Anthocyanidins in Bilberry Extracts. J. Agric. Food Chem..

[42] Yu H., Wang J., Zhao Z., Sheng X., Shen Y., Branca F., Gu H. (2019). Construction of a high-density genetic map and identification of loci related to *hollow stem trait* in broccoli (*Brassic oleracea* L. *italica*). Front. Plant Sci..

[43] Li H., Durbin R. (2009). Fast and accurate short read alignment with Burrows-Wheeler Transform. Bioinformatics.

